# Viral Encephalitis of Unknown Cause: Current Perspective and Recent Advances

**DOI:** 10.3390/v9060138

**Published:** 2017-06-06

**Authors:** Peter G. E. Kennedy, Phenix-Lan Quan, W. Ian Lipkin

**Affiliations:** 1 Department of Neurology, Institute of Neurological Sciences, Glasgow University, Southern General Hospital, Glasgow G51 4TF, UK; 2 Center for Infection and Immunity, Mailman School of Public Health, Columbia University, 722 W 168th Street, New York, NY 10032, USA; pq2106@cumc.columbia.edu (P.-L.Q.); wil2001@columbia.edu (W.I.L.)

**Keywords:** virus, encephalitis, virus discovery, unbiased high-throughput sequencing

## Abstract

Viral encephalitis causes acute inflammation of the brain parenchyma and is a significant cause of human morbidity and mortality. Although Herpes Simplex encephalitis is the most frequent known cause of fatal sporadic encephalitis in humans, an increasingly wide range of viruses and other microbial pathogens are implicated. Up to 60% of cases of presumed viral encephalitis remain unexplained due to the failure of conventional laboratory techniques to detect an infectious agent. High-throughput DNA sequencing technologies have the potential to detect any microbial nucleic acid present in a biological specimen without any prior knowledge of the target sequence. While there remain challenges intrinsic to these technologies, they have great promise in virus discovery in unexplained encephalitis.

## 1. Introduction

Encephalitis refers to inflammation of the brain parenchyma that typically presents with a combination of features including fever, headache, clouding of consciousness, seizures, personality change, focal neurological deficits, and coma [1,2]. It is often accompanied by meningitis and inflammation of the membranes that cover the brain and spinal cord. Encephalitis must always be distinguished from an encephalopathy, which may present similarly but results from metabolic disturbances caused by liver or renal failure, intoxications, systemic infections, or anoxia, rather than from inflammation [2,3].

Forty to sixty percent of encephalitis cases are unexplained [1,2,4,5]. Encephalitis can be caused by direct infection by microbial agents (bacteria, fungi, viruses, parasites) or can be immune-mediated [1,3,6]. Immune-mediated encephalitis usually occurs as a result of a post-infectious process such as acute disseminated encephalomyelitis (ADEM), typically following infection with measles, mumps, or rubella virus infection [6,7], or, as has more recently been increasingly described, due to antibody-mediated encephalitis such as those caused by antibodies to voltage-gated potassium channels or to the *N*-methyl-d-aspartate (NMDA) receptor [8,9].

Viruses are an important, yet often poorly understood cause of encephalitis. A range of viral agents have been implicated [10]. Herpes Simplex virus (HSV)-1 is the virus most commonly implicated in fatal sporadic encephalitis; however, other viruses implicated in encephalitis include herpesviruses (HSV-2, varicella-zoster, cytomegalovirus, Epstein-Barr, and HHV 6 and 7); paramyxoviruses (measles, rubella); orthomyxoviruses (influenza A virus); enteroviruses (EV 70 and 71, polio-, echo- and coxsackieviruses); flaviviruses (West Nile, Japanese encephalitis, dengue and Zika viruses); retroviruses (human immunodeficiency virus); alphaviruses (Venezuelan equine-, eastern equine-, western equine-encephalitis); bunyaviruses (La Crosse virus); rhabdoviruses (rabies virus); parvovirus (B19); and astroviruses [3,6,11].

## 2. Differential Diagnosis of Presumed Viral Encephalitis

The most relevant means of neurological identification of a causative agent in encephalitis is by examination of the cerebrospinal fluid (CSF) or brain tissue obtained at either brain biopsy or autopsy. Methods employed include virus isolation, polymerase chain reaction (PCR), immunohistochemistry or electron microscopy, serology, and, more recently, high-throughput sequencing (HTS). Viral isolation is the classical method for diagnosis, but it is increasingly used in concert with PCR, which has become more accessible and can provide near immediate actionable data. Culture requires days to weeks and may fail in the event that the virus cannot be propagated in the cell line selected, whereas PCR requires only a few hours.

The role of CSF PCR in the diagnosis of viral infections of the nervous system has been extensively reviewed [12]. While the specificity and sensitivity of some PCR assays such as that for HSV-1 DNA are exceptionally high at 96% and 99% respectively, other assays are not as robust. For example, while it was originally thought that PCR for central nervous system (CNS) enteroviral infections was very sensitive at 95%, more recent data suggest that this figure may be considerably lower [12]. Because of the various potential problems with PCR in neurovirological diagnosis, caution must be exercised when basing the identification of novel CNS viruses with this technique alone.

Serology can succeed where both culture and PCR fail; however, diagnosis may require the collection of samples at two different time points when IgM is negative, and cross-reactive antibodies can confound specificity, as during the early phase of investigation during the 1999 West Nile virus outbreak in North America when St. Louis encephalitis virus was inaccurately implicated [13,14]. Furthermore, elevated antiviral antibodies may simply represent nonspecific polyclonal activation of memory B cells from a previous infection [15].

Diagnosis of an infection is best achieved by obtaining a specimen directly at the site of disease. However, a brain biopsy is often a measure of last resort and, consequently, the specimen is rarely available. When accessible, the scarce amount of tissue material available greatly restricts the number of experiments that can be performed. In most cases, CSF is the only surrogate specimen available for the assessment of CNS disorders.

In instances where CNS sampling is not possible or fails to provide clues to a causative agent, some clinicians and investigator survey blood, the oropharynx, feces, or urine. The identification of an agent in any sample type may lead to targeted assays of CSF or antibody tests, if seroconversion is consistent with acute infection.

## 3. Viral Discovery

The advent of unbiased DNA sequencing technologies offers unprecedented opportunities to detect unexpected, previously unrecognized, or novel microbial pathogens in undiagnosed encephalitis. Unlike sequence-based methods that are limited to the detection of known or related agents, unbiased high-throughput sequencing (HTS) has the potential to detect any microbial nucleic acid present in a biological specimen without any prior knowledge of the target sequence. The ability of sample multiplexing using barcodes enables simultaneous sequencing of a large number of samples in a single run. This strategy fully exploits HTS systems by increasing throughput while reducing sequencing costs [16].

## 4. Challenges in High-Throughput DNA Sequencing

Sample collection, transporting, handling, and storage conditions can profoundly affect the quality of the sample and thus sequencing results. The development of laboratory standard operating procedures is critical to ensure high quality input material.

While HTS provides the most broad-based, unbiased approach for the detection of known and novel viral agents from biological samples, it also faces challenges related to sample preparation and data analysis that impede widespread use of the technique. The sample preparation protocols for HTS require the removal of host-derived sequences, which are present in the sample, in order to ensure that the sequence reads are of viral rather than host origin. Additionally, the massive amount of sequence information generated in HTS requires extensive support from bioinformatics experts, customized bioinformatic tools and powerful computer clusters to efficiently and rapidly analyze the data.

The choice of a sample preparation protocol strictly depends on the nature of the biological specimen. Because host nucleic acid is present in great abundance as compared to virus genetic material in biological specimens, procedures such as the depletion of host background or the enrichment of viral genomes is critical to ensure the threshold detection of a viral signature. This is particularly true for complex clinical specimens such as tissue samples like brain [17]. Conversely, cell-free samples such as CSF contain low levels of starting material and specimens should first be filtered or centrifuged to reduce the volume of the sample and to maximize the isolation of nucleic acids [17].

Viral nucleic acids may be enriched using either subtractive or positive selection methods. Host and non-viral templates can be reduced through filtration, ribosomal RNA subtraction, and nuclease treatment [17,18,19,20,21]. Filtration is particularly useful for the analysis of fecal samples and has become standard practice, irrespective of whether additional enrichment strategies are employed [21]. Ribosomal rRNA subtraction has the potential to remove 95% of the host RNA; however, this level of efficiency is rarely achieved [20]. Nuclease treatment is the most powerful of the subtractive methods [18]. The rationale in this approach is that viral nucleic acid should be protected within its nucleocapsid. However, in the event that the viral template is not contained within a nucleocapsid, it will be lost to analysis. Thus, we cannot detect intracellular viral RNA using nuclease treatment.

The systematic analysis of extensive sequence data requires customized bioinformatic tools as well as powerful computers. Genomic footprints of viruses are detected using Basic Local Alignment Search Tool (BLAST) programs that compare sequences obtained by sequencing to those present in a reference database. This sequence-based approach relies on the concept that new viruses will share homology with known viruses.

The pipeline starts with the trimming of raw sequence reads to remove the adapter sequences from the reads. Sequences are then filtered to eliminate low-complexity reads and repetitive sequences. The dataset is further enriched for potential viral sequences by removing contaminating sequence reads derived from host and bacterial sequences, followed by the clustering of redundant sequences. The remaining sequences are further assembled into contiguous sequences and compared to the GenBank databases at the nucleotide (BLASTN) and to the non-redundant protein sequences (nr) database from NCBI (http://www.ncbi.nlm.nih.gov) using tBLASTx and BLASTx.

## 5. VirCapSeq-VERT

More recently, a positive selection method analogous to exon capture for viruses has been established for viral diagnosis and discovery (VirCapSeq-VERT) [22]. The approach entails enriching for relevant targets prior to analysis using probes that represent genomic sequences of known viruses. VirCapSeq-VERT has been shown to have sensitivity similar to PCR and is less cumbersome than enrichment by filtration and nuclease treatment [22].

**Figure 1:**
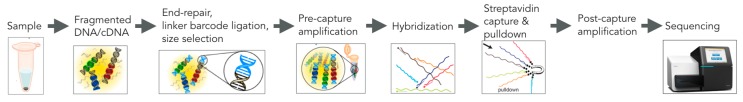
VirCapSeq-VERT target enrichment.

The system employs approximately 2 million biotinylated oligonucleotide probes designed to bind to the coding sequences of all viral taxa known to infect vertebrates at intervals of 50–100 nt. Libraries prepared from random primed cDNA are hybridized with the biotinylated VirCap probes. Streptavidin magnetic beads are added to trap biotinylated probes and their complementary library components. After magnetic capture and washing, nucleic acid is released from the beads and subjected to post-hybridization PCR prior to sequencing (Figure 1). Demultiplexed filtered reads are compared by BLASTn to reference databases to remove the host background, assembled and then subjected to a homology search in GenBank nucleotide and protein databases. This method yields a 100- to 10,000-fold increase in viral reads from serum, blood, feces, saliva, and tissue homogenates compared to conventional Illumina sequencing using established virus enrichment procedures, including filtration, nuclease treatments, and RiboZero rRNA subtraction. This reduces the number of reads required per sample from approximately 200 M to 5 M and streamlines bioinformatic analysis. VirCapSeq-VERT has a limit of detection comparable to that of agent-specific real-time PCR.

A potential confounding factor is that VirCapSeq-VERT may not detect entirely new viruses; however, the likelihood of missing a virus that causes encephalitis is low because probe nucleic acid homology to target is not as stringent as PCR; the entire genomic sequence of each virus is accessible for capture and databases and probe libraries are continuously updated.

## 6. Microbial Contamination in HTS

The introduction of contaminating microbial sequences during the experimental process will lead to false-positive findings and erroneous data interpretation [23,24,25,26]. It may also be a confounding factor in the assessment of complex clinical samples such as tissue samples, cell-free samples, and samples where an agent is generally present at low levels [23].

The identification of *bona fide* microbial contaminations may be identified by systematically processing and sequencing a mock library preparation and a control sample in parallel with the experimental samples. Additionally, the use of a repository of contaminating sequences may help in some cases alleviate the effects of contamination by computationally excluding them from downstream analysis. Importantly, any microbial signature identified by HTS needs to further be validated in the original sample by specific PCR assays based on newly identified microbial genome sequences.

## 7. A Diagnostic Algorithm for Encephalitis

**Figure 2:**
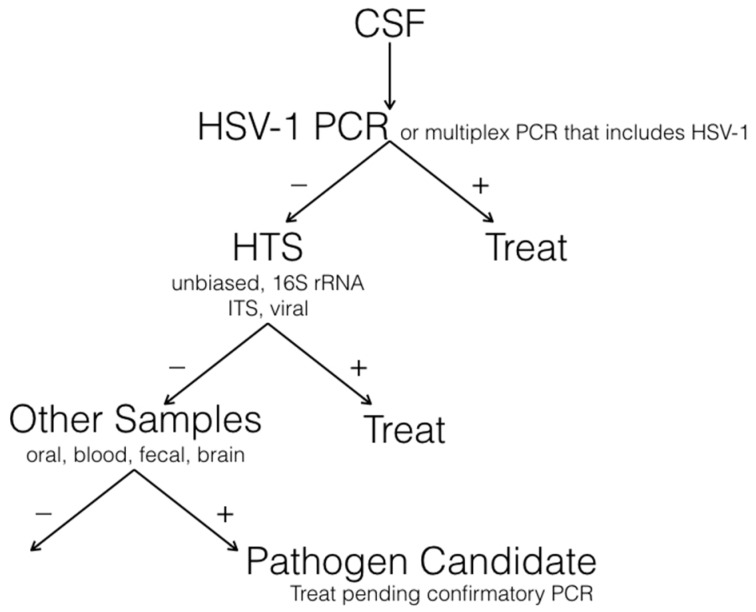
Strategy for laboratory investigation of unexplained encephalitis.

A suggested flow chart for the laboratory investigation of unexplained viral encephalitis is shown in Figure 2. The ideal sample source is brain tissue; however, brain biopsies are typically reserved for cases where a diagnosis cannot be made from CSF or the patient does not respond to empirical therapy. CSF is screened first for HSV-1, the virus most commonly implicated, using single-agent or multiplex real-time PCR. These assays can be completed in a few hours and have the potential to detect less than 10 genome copies [22]. Current dye technology readily supports up to five different gene targets. We typically reserve one for a housekeeping gene (to ensure the presence of a nucleic acid template in the sample as well as the integrity of the assay) and use the remaining four for microbial targets. By assembling several panels, we can rapidly test for the presence of 20 or more agents [27].

The presence of a signal provides an initial guide for therapy; however, CSF should be retested to ensure the validity of a finding. Cell counts and differential cell counts, as well as protein and glucose measures may be helpful; nonetheless, we proceed with comprehensive molecular analyses until a specific diagnosis is achieved, irrespective of whether the pattern is more consistent with bacterial or viral infection. Where PCR fails to implicate an agent, we move to high-throughput sequencing of CSF. High-throughput sequencing techniques have the potential to detect nucleic acids that represent viruses, bacteria, fungi, or parasites. However, the method is less sensitive than the analysis of libraries that target specific phyla. Accordingly, we typically sequence libraries prepared following 16S rRNA (bacteria) [28], internal transcribed spacer (ITS, fungi) region amplification [29,30], or selection using biotinylated probes to viruses (VirCapSeq-VERT). In laboratories where sequencing is routine, the time required from sample receipt to bioinformatics analysis is less than 72 h. Where high-throughput sequencing of CSF fails, we recommend proceeding directly to brain biopsy; however, an alternative strategy is to search other sites and samples including the oropharynx (oral swabs), gastrointestinal tract (feces), urine, or blood for the presence of potential pathogens that might have metastasized in the brain.

## 8. Examples of HTS in Unexplained Viral Encephalitis

Unbiased HTS has proven to be successful in detecting infectious agents in cases of encephalitis of unknown etiology, including a novel lymphocytic choriomeningitis virus-related arenavirus that caused transplant-associated encephalopathy and an astrovirus as the cause of fatal encephalitis in an immunocompromised patient [11,31]. These novel viruses were only identified by HTS after all conventional diagnostic methods including PCR, serology, and oligonucleotide microarray analyses had failed to identify an infectious agent. Following this, specific PCR assays based on newly identified viral genome sequences as well as immunohistochemical or serological assays were used to confirm the infections. The case of the immunocompromised patient with the astrovirus encephalitis illustrates the power of HTS in identifying a novel and unexpected virus not previously associated with a CNS infection. Astroviruses are being increasingly recognized as significant neurotropic pathogens in immunocompromised patients [32,33,34,35].

Furthermore, recent studies have identified both Parvovirus 4 (PARV4), an emerging virus identified recently in human blood and other tissues with no known associated disease, and a novel cyclovirus in CSF specimens from patients with acute central nervous system infections of unknown etiology [36,37].

## 9. Causal Relationship to Disease

This last point reinforces the notion that the identification of viral sequences in clinical material is not necessarily the same as showing a causal association between the virus and the neurological disease. Such an association is easiest to make when the infectious agent has been isolated at an appropriate site during the course of active disease. However, access to specimens such as brain biopsy is generally not available and the only surrogate specimen available for analysis is CSF. The possibility always exists that the novel virus identified may play no role in disease pathogenesis and represents an irrelevant co-infection. We must always consider and control for the possibility of sample contamination, a particular hazard with PCR (especially nested-PCR). Nevertheless, identification of a virus in a case of presumed viral encephalitis would normally be construed as strong evidence of causation unless there was additionally strong evidence to suggest either accidental contamination during the procedure or an irrelevant co-existing infection.

Since CSF is normally acellular and sterile, clear evidence of a microbial signature in CSF merits strong consideration as a candidate pathogen. Failure to detect a viral agent in a CNS disease such as viral encephalitis may reflect the absence of its molecular footprint at the time of sampling. Notwithstanding these caveats, investigating CNS infections of unknown etiology using unbiased HTS or VirCapSeq-VERT may identify the complete spectrum of causative agents associated with encephalitis. Further, early recognition of the causative agent of unexplained encephalitis cases could enable specific interventions that reduce illness and death and facilitate the recognition of outbreaks that threaten public health.

## 10. Conclusions

Despite the progress that has been made with both classical virological and molecular biological technologies in identifying the numerous causes of viral encephalitis, there is an increasing need for better tools and new approaches for detecting both genuinely novel pathogens in such patients and determining new manifestations of known pathogens, including viruses. In evaluating the results of different studies, it is crucial to have uniform clinical and diagnostic criteria for establishing a definite diagnosis of viral encephalitis. There is not a standardized algorithm for the molecular investigation of unexplained encephalitis, though general guidelines for the investigation and management of suspected viral encephalitis are now available [6,38]. Problems of contamination of tissues during procedures, and adequate sensitivity and specificity continue to influence the interpretation of CSF PCR. High-throughput sequencing technology has added a new and exciting dimension to our ability to unambiguously identify the presence of novel viruses in patients with encephalitis of unknown cause. Nevertheless, caution must always be exercised in assuming a direct cause and effect relation between the newly identified virus and the neurological infection. The extent to which the anticipated identification of novel viruses in viral encephalitis will lead to direct and early improvement in new therapies and a better prognosis for such individuals is difficult to predict. However, these advanced genomic techniques certainly have great potential in improving the diagnosis and management of patients with a range of viral infections [39]. It is possible that the initial benefits may be more applicable to immunocompromised individuals with CNS viral infections, in whom novel viral infections may be easier to detect.
